# Tuning the photoexcitation response of cyanobacterial Photosystem I via reconstitution into Proteoliposomes

**DOI:** 10.1038/s41598-017-02746-5

**Published:** 2017-05-30

**Authors:** Hanieh Niroomand, Dibyendu Mukherjee, Bamin Khomami

**Affiliations:** 1 Sustainable Energy Education and Research Center (SEERC), Knoxville, USA; 2 Department of Chemical and Biomolecular Engineering, Knoxville, USA; 3 Department of Mechanical, Aerospace and Biomedical Engineering, University of Tennessee, Knoxville, USA

## Abstract

The role of natural thylakoid membrane housing of Photosystem I (PSI), the transmembrane photosynthetic protein, in its robust photoactivated charge separation with near unity quantum efficiency is not fundamentally understood. To this end, incorporation of suitable protein scaffolds for PSI incorporation is of great scientific and device manufacturing interest. Areas of interest include solid state bioelectronics, and photoelectrochemical devices that require bio-abio interfaces that do not compromise the photoactivity and photostability of PSI. Therefore, the surfactant-induced membrane solubilization of a negatively charged phospholipid (DPhPG) with the motivation of creating biomimetic reconstructs of PSI reconstitution in DPhPG liposomes is studied. Specifically, a simple yet elegant method for incorporation of PSI trimeric complexes into DPhPG bilayer membranes that mimic the natural thylakoid membrane housing of PSI is introduced. The efficacy of this method is demonstrated via absorption and fluorescence spectroscopy measurements as well as direct visualization using atomic force microscopy. This study provides direct evidence that PSI confinements in synthetic lipid scaffolds can be used for tuning the photoexcitation characteristics of PSI. Hence, it paves the way for development of fundamental understanding of microenvironment alterations on photochemical response of light activated membrane proteins.

## Introduction

The quest for green energy has sparked considerable interest in Photosystem I (PSI), the photosynthetic protein complex, that is akin to a nano-scale biological photodiode and enables light-activated charge separation (with nearly 100% quantum efficiency) to facilitate unidirectional electron flow^1^. The robust structural and photoactive electrochemical properties of PSI, a transmembrane protein, makes it an ideal candidate for incorporation into solid state bioelectronic or hybrid photovoltaic devices^2^. But the first step towards the successful fabrication of such bio-hybrid devices call for systematic assembly of oriented and functional PSI onto desired bio-abio interfaces via suitable protein scaffoldings. To this end, one must address the obvious question regarding the role of the natural thylakoid membrane housing of PSI trimeric complex in providing the required structural and functional scaffold to the protein. This had led to a growing interest in membrane reconstitution of PSI complexes to form stable proteoliposomes as the final product. Reconstituted proteoliposomes serve as experimental systems for the study of membrane enzymes^3^ and small helical membrane proteins, providing a sample environment that accurately mimics the native membrane environment and properties such as hydrophobic thickness, water concentration gradient and lipid order parameter gradient^4, 5^. Hence, our motivation for the present study stems from the desire to attain systematic incorporation of PSI complexes into synthetic membrane-bound structures that mimic the natural thylakoid membrane housing of PSI.

3D structures of several membrane proteins such as bacteriorhodopsin^6^, aquaporin^7^, EmrE^8^ have been determined using electron crystallography. However, both interactions within the protein as well as between the protein and its environment, influence the protein structure^9^. Thus, synthetic lipid bilayers have been employed as biomimetic environments to enable the structural characterizations of several membrane proteins such as, Gramicidin A^10^, M2 protein from the influenza A virus^11, 12^, trimeric structures from membrane-bound proteins involved with eicosanoid and glutathione metabolism^13, 14^, and the mercury transporter MerF^15^. Crystallographic studies of PSI at 2.5 A° resolution have identified four lipids consisting of three phosphatidylglycerol (PG) molecules and one monogalactosyldiacylglycerol (MGDG) molecule to be the integral cofactors of the transmembrane parts of PSI^16^. These lipids are considered to be largely responsible for the long-term stability and functionality of PSI^17^. In light of the large negatively charged PG content in PSI lipid structures, it becomes pertinent to design bio-mimetic membranes with high PG lipid contents that can be used for large membrane protein reconstitution.

In the past, our research group has performed successful photocurrent measurements on colloidal chemistry-driven and directed assembly of detergent-bound PSI monolayers on self-assembled monolayer (SAM) substrates^2, 18–20^. In spite of these efforts, the relatively low levels of photocurrent generation from these systems has increasingly drawn our attention towards the role of natural membrane scaffoldings in PSI trimeric complex in enhancing the efficiency, stability and lifetime of its photochemistry. Recently, Saboe *et al*. reconstituted 2D PSI crystals on a tethered bilayer lipid membrane support with intercalated conjugated oligoelectrolyte (COE) units^21^. In another work, Stieger *et al*. constructed 3D architectures consisting of the redox protein cytochrome c as a molecular scaffold for PSI as the photo-functional matrix element aided by DNA molecules as further building blocks^22^. Both of these studies show significant photocurrent enhancements due to the incorporation and possibly, the confinement of PSI in a biomimetic scaffold. These promising results shed light on the photocurrent enhancements arising from the high packing density and orientation of PSI in these complexly tailored biomimetic scaffolds. Yet, the exact role of the specific membrane scaffolds in driving the enhanced photoactive functionality and near unity quantum efficiency in PSI owing to the conformational changes in its native membrane bound form remains elusive.

Furthermore, a recent work on Photosystem II (PSII) integrated into electrodes, revealed that photosystems lacking the membrane environment undergo short circuiting electron transfer between electrode materials and the surface exposed chlorophylls^23^. This competing charge transfer pathway through the chlorophylls of the light-harvesting antenna results in underperformance of these integrated bio-photoelectrochemical systems^24^. It was additionally proposed that this short circuiting induces the generation of toxic free chlorophyll, and in turn the production of reactive oxygen species (ROS). ROS produced at the antenna chlorophylls might promote rapid deactivation of the photosynthetic proteins^23^. While very fast deactivation was shown for PSII when integrated in devices (half-life as low as 4 min when PSII is wired to electrodes via redox-active polymers)^25^ the same effect can be anticipated for PSI-based systems too. Indeed, half-life as low as 15 min was reported for the normally highly robust PSI protein upon integration in electron conducting matrices (redox hydrogel film) in absence of any lipid membrane^26^. Hence, one of the outlooks for the rational design of pigment–containing systems require strategies involving synthetic lipid membranes, as well-defined protein–pigment scaffolds, that can ensure insulation from unwanted charge transfer processes and control dissipation of excitation energy to minimize competing reactions with oxygen.

In our continual effort to investigate the optoelectronic behaviors of PSI confined under different bio-abio interfaces, this study presents a fast and elegant approach to achieve high density PSI encapsulation in synthetic lipid bilayer membranes to constitute PSI-proteoliposomes. To this end, we have recently investigated the phase transitions during detergent mediated solubilization of negatively charged 1,2-dipalmitoyl-*sn*-glycero-3-phospho-(1′-*rac*-glycerol) (DPPG) liposomes, a member of the PG lipid family. The highly irregular structural arrangements that arises in this system at room temperature indicates its unsuitability for successful protein insertion. Furthermore, this study unveiled the possibility of 1,2-diphytanoyl-*sn*-glycero-3-phosphocholine (DPhPC) as a promising lipid candidate for protein insertions^27^. It should be noted that DPhPC is a branched phosphatidylcholine (PC) lipid with high thermal and structural stability^28^.

A significant amount of data has been amassed regarding the binary^29, 30^ and ternary lipid mixtures^30, 31^ including the miscibility phase diagrams for several of these systems. However, the majority of these studies have been performed for systems that contain only uncharged lipids. Miscibility studies on lipid mixtures containing charged lipids, in particular binary^32, 33^ and ternary mixtures of PC with PG^34^ have shown that membranes containing charged PG lipids indicate similar phase behavior as membranes containing only uncharged PC lipids. Considering the predominant presence of PG in the natural thylakoid membrane of PSI, we introduce a PG-based lipid as our choice for the study of reconstituted PSI proteins. To this end, in the present study we have chosen to work with DPhPG (1,2-diphytanoyl-*sn*-glycero-3-phospho-(1′-*rac*-glycerol)), a lipid with similar structure and thermotropic state that corresponds to the transition temperature of DPhPC. DPhPG is an anionic lipid with 16-carbon long hydrocarbon chains that are methyl-branched, stable and in fluid state over wide temperature ranges.

**Figure 1:**
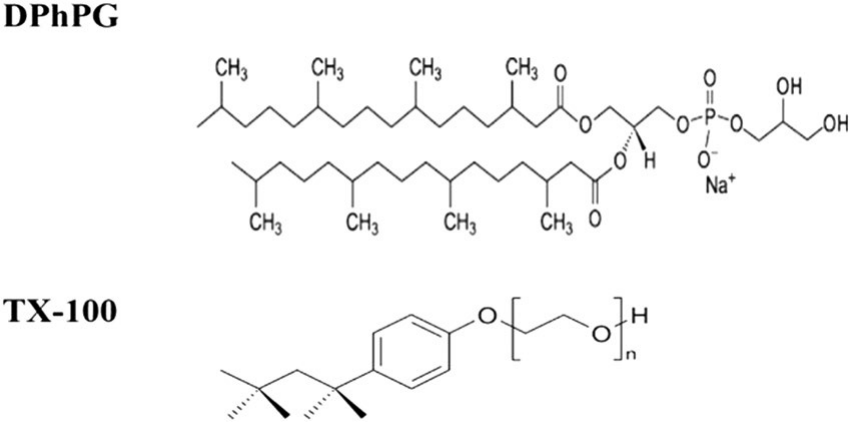
Molecular structure of lipid (DPhPG) and detergent (TX-100). DPhPG contains two hydrocarbon chains of 16 carbons with total lipid molecule length of ~2–3 nm. TX-100 molecule length is ~3.75–4.0 nm.

The current work involves the systematic protein incorporation into DPhPG liposomes. Four basic strategies can be used for the insertion of membrane proteins into liposomes. These include sonication, freeze-thawing, organic solvents, and detergents. The first three methods have significant limitations in efficient proteoliposome reconstitutions that include possible local probe heating during sonication that leads to degradation and denaturation of many membrane proteins. Similarly, organic solvents denature most amphiphilic membrane proteins even though reverse-phase evaporation and solvent injection using organic solvents enable efficient liposome preparations^35, 36^. However, since detergents are regularly employed during membrane protein purification processes; hence, detergent mediated solublization has become a preferred route for proteoliposome preparations^37^. To this end, fundamental understanding of the solution phase morphology of membranes during their interactions with detergent that determines the all-important membrane-detergent phase diagrams becomes critical for successful isolation, purification, reconstitution and crystallization of membrane proteins^38–40^. Thus, we first investigated the surfactant-induced membrane solubilization of DPhPG by a prototypical non-ionic detergent Triton X-100 (TX-100). Here, for ease of visualization and understanding, we have shown the structural details of both DPhPG and the detergent TX-100 in Fig. 1.

Isothermal titration calorimetry (ITC) has been classically used as a robust analytical technique for various application such as measuring binding affinities of ligands to membrane proteins^41^, optimizing inhibitor binding in drug discovery^42^, and accessing the partitioning of surfactants into electrically neutral lipid bilayer membranes^43^. In general, ITC is the preferred method for studying interactions between a bioactive molecule (surfactant/peptide/protein) and model lipid membranes^44–48^. In the present work, we have utilized ITC measurements for detecting composition-dependent transitions in lipid-detergent systems. Furthermore, dynamic light scattering (DLS) and cryo-transmission electron microscopy (cryo-TEM) imaging techniques are used to monitor the different stages of stepwise solubilization of liposomes with varying detergent concentrations. Specifically, the composition-dependent equilibrium transitions (phase diagram) of lipid/detergent mixtures is elucidated. Based on previous studies that indicate the occurrence of most successful protein incorporations during the second stage of detergent-mediated solubilization^49–52^, our prior studies set the backdrop for ideal detergent/lipid concentration ratios for successful protein insertions.

The latter part of our studies will utilize the aforementioned phase diagrams to achieve systematic incorporation of PSI into detergent-destabilized preformed-liposomes at the second stage of solubilization upon subsequent adsorption of detergents using polystyrene beads^9, 30, 41^. Size-exclusion chromatography (SEC) using high resolution fractionation based on size differences of various complexes has been employed to assure the partitioning of proteoliposomes from individual proteins and/or, excess detergents. Finally, we will confirm the PSI-proteoliposome formation via absorption and fluorescence spectroscopy measurements as well as high-resolution AFM imaging to directly visualize the proteoliposomes supported on gold substrates.

## Results

This section details our major results for the PSI-proteoliposome formation with the DPhPG/TX-100/PSI system, which can be briefly summarized as follows: (1) Phase diagrams of surfactant induced DPhPG solubilization; (2) Reconstitution of PSI trimers in DPhPG liposomes analyzed with the aid of absorption spectroscopy, fluorescence measurements and atomic force microscopy imaging.

### Surfactant Induced DPhPG Solubilization

**Figure 2:**
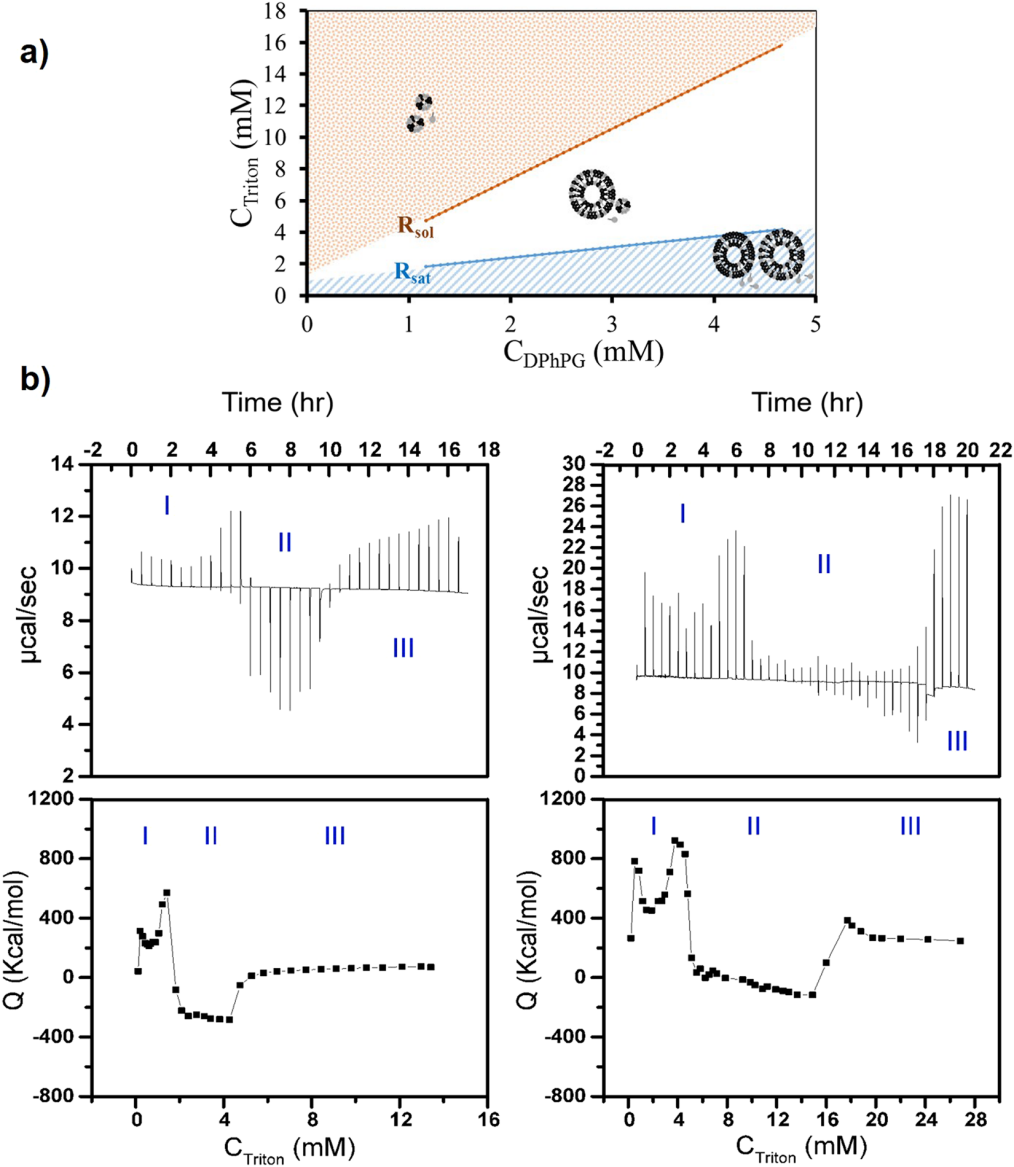
Solubilization of preformed DPhPG liposomes induced by stepwise increase of TX-100 concentration. (**a**) phase diagram of DPhPG/TX-100 mixture in buffer; (**b**, left panel) solubilization titration of 1 mg ml^−1^ DPhPG liposomes with 77 mM TX-100, Compensation heat power, Δ*p*, versus time, *t*, on top and heats of injection, Q, as a function of the total detergent concentration, C_Trit_, on bottom; (**b**, right panel) solubilization titration of 4 mg ml^−1^ DPhPG liposomes with 154 mM TX-100, Compensation heat power versus time on top and heats of injection as a function of the total detergent concentration on bottom.

Membrane solubilization typically comprises a three-stage process represented by the structural transitions that occur as detergents interact with lipid membranes^39, 53–55^. As a function of increasing detergent concentrations, the aforementioned stages are: (I) the stage containing detergent monomers and vesicles wherein detergent monomers start getting incorporated into vesicles; (II) the stage where detergent monomers, mixed vesicles, and mixed micelles coexist (the saturated boundary indicated as R_sat_ in Fig. 2a); and (III) the final stage that constitutes pure micelles at varying detergent/lipid ratios and detergent monomers (the solubilization boundary indicate as R_sol_ in Fig. 2a). These three stages of detergent-liposome interactions are identified as a function of increasing detergent concentrations from ITC measurements and depicted in the phase diagrams in Fig. 2a and b.

Phase diagram of DPhPG/TX-100 mixture in 200 mM Na-Phosphate buffer is shown in Fig. 2a. Data are collected from two series of ITC solubilization experiments starting with two different lipid concentrations (~1 mg ml^−1^ and 4 mg ml^−1^) wherein the R_sat_ and R_sol_ boundaries are as indicated. (Refer to Supplementary Material for complete phase diagram analysis). Figure 2b (top left panel) represents the raw data from the titration of 1 mg ml^−1^ DPhPG vesicles with 77 mM of TX-100 in buffer at room temperature. The three stages of detergent-liposome interactions are identified as vesicles (I), coexistence (II) and micelles (III) in the curve of compensation heat power, Δ*p* (μcal/sec) versus time, *t*. Figure 2b (bottom left panel) represents the heats of injection, Q (kcal/mol) as a function of increasing TX-100 concentrations, C_Trit_ (mM). Figure 2b (top and bottom right panel) represent Δ*p* versus time *t*, and Q as a function of increasing C_Trit_ resulting from the integration of the power peaks obtained from the titrations of 4 mg ml^−1^ DPhPG vesicles with 154 mM TX-100, respectively. The onset of solubilization (*R*_sat_) is represented by the drop in Q (exothermic reaction at stage I – II transition), followed by a region of virtually constant Q (stage II). Beyond this region, an increase in Q (stage II-III transition) marks the completion of solubilization (*R*_sol_). The *R*_sat_ and *R*_sol_ breakpoints read from these curves are included in Fig. 2a. The significant findings from our solubilization studies of DPhPG with TX-100, namely, the saturation (R_sat_) and solubilization (R_sol_) concentrations of TX-100 are summarized in Table 1.

**Table 1:** Highlights of lipid/detergent concentrations, saturation (R_sat_) and solubilization (R_sol_) concentrations from TX-100 induced DPhPG solubilization.

Lipid Concentration (mg/ml)	Detergent Concentration (mM)	R_sat_ (mM)	R_sol_ (mM)
1	77	1.83	4.75
4	154	4.19	15.85

**Figure 3:**
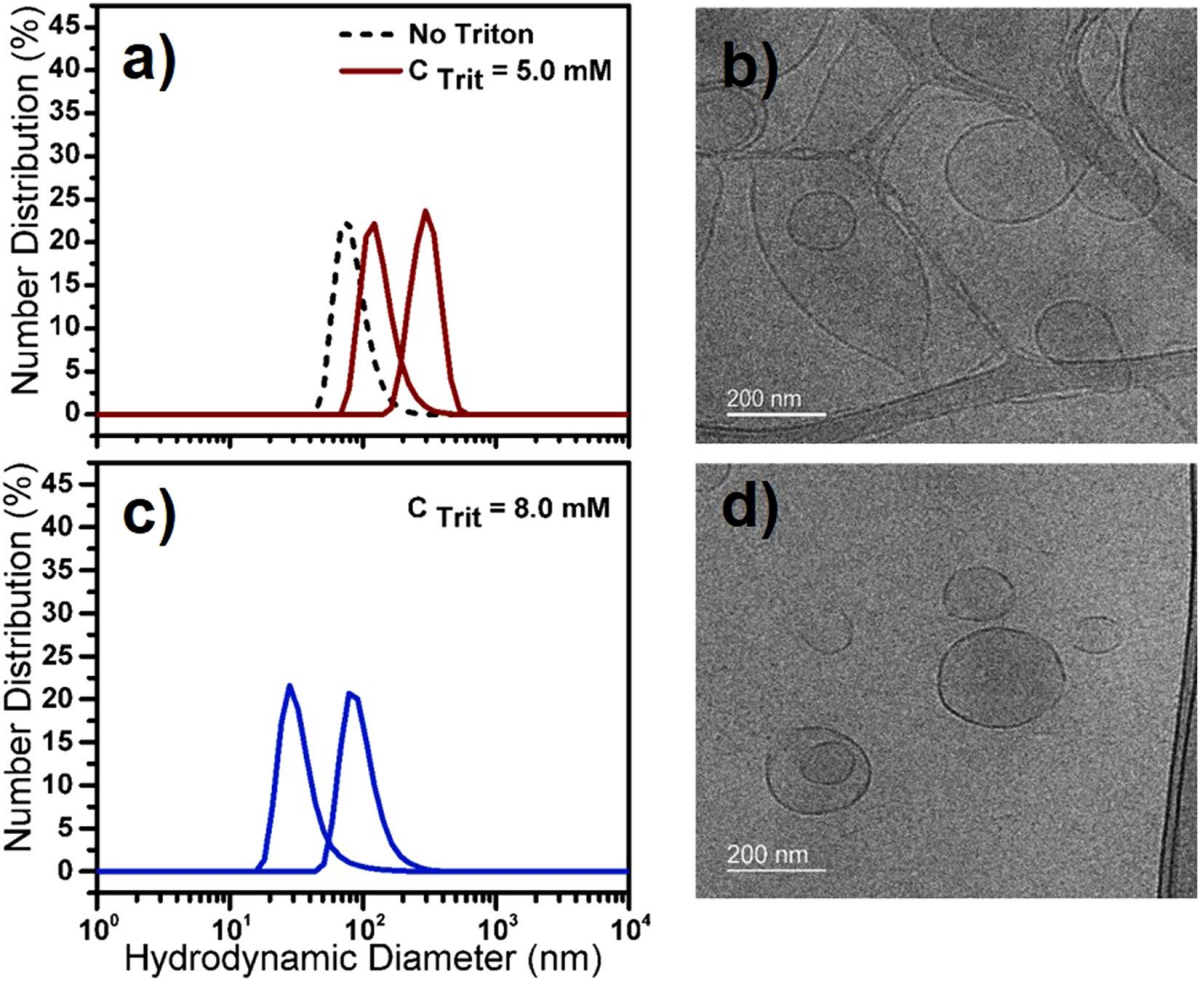
Number distribution (%) from DLS measurements (**a**,**c**) and corresponding cryo-TEM micrographs (**b**,**d**) of preformed DPhPG liposomes during the second stage (stage II) of solubilization with TX-100. Liposomes were treated with different TX-100 concentrations of (**a**,**b**) C_Trit_ = 5.0 mM, and (**c**,**d**) C_Trit_ = 8.0 mM.

Size and morphological variations during the stage II of solubilization are elucidated with cryo-TEM images of liposome structures in their vitrified states (Fig. 3b and d) as well as DLS measurements (Fig. 3a and c). Cryo-TEM images for 4 mg mL^−1^ DPhPG liposome solubilization with detergent concentrations ranging between *R*_sat_ < C_Trit_ < *R*_sol_ indicate the enlargement in the liposome diameter due to detergent incorporation within the membrane bilayers. Particle size distributions (PSD in terms of number distribution, %) corresponding to solubilization with C_Trit_ = 5 mM indicate two peaks at ~150 and 320 nm as compared to the peak size ~100 nm for liposomes without any TX-100 (Fig. 3a). We also observe that these enlarged liposomes coexist with stable open vesicular structures at C_Trit_ ~ 5.0 mM (see Fig. 3b). This bilayer opening is associated with the rapid solubilization of bilayers by detergents with relatively small polar moiety as in the case of TX-100. Such rapid solubilization is achieved due to detergent molecules interacting from both sides of the phospholipid bilayer (transbilayer solubilization) that ultimately makes it permeable and in turn disintegrates into mixed micelles^56–59^. Upon further increase of detergent concentrations (C_Trit_ ~ 8.0 mM), enlarged vesicles continue to disintegrate into mixed micelles and open vesicular structures with smaller size (~30–90 nm) as observed from both DLS measurements (Fig. 3c) and cryo-TEM micrographs (Fig. 3d). Based on these data, C_Trit_ concentrations were chosen from points within the coexistence region (stage II) of the solubilization phase diagrams in Fig. 2a to execute our PSI reconstitutions in the liposomes.

### Liposome-Based Reconstitution of Trimeric PSI

PSI-proteoliposme reconstitutions were carried out by incorporating TX-100 solubilized PSIs into TX-100 destabilized liposomes at multiple points chosen from within the stage II of solubilization. Following the PSI insertion, the excess detergents were adsorbed by using polystyrene beads. Two points within the stage II of solubilization were chosen: C_Trit_ = 8.0 mM (intermediate part of stage II), and C_Trit_ = 12.0 mM (end of stage II). Protein reconstitution was performed at three weight protein-to-lipid ratios (wPLR) of 0.26, 0.53 and 1.2. Subsequently, size-exclusion chromatography (SEC) fractions were collected prior to any PSI-proteoliposome analysis whereby PSI-proteoliposmes were separated from individual PSIs, small aggregates or excess detergent based on their physical size difference.

**Figure 4:**
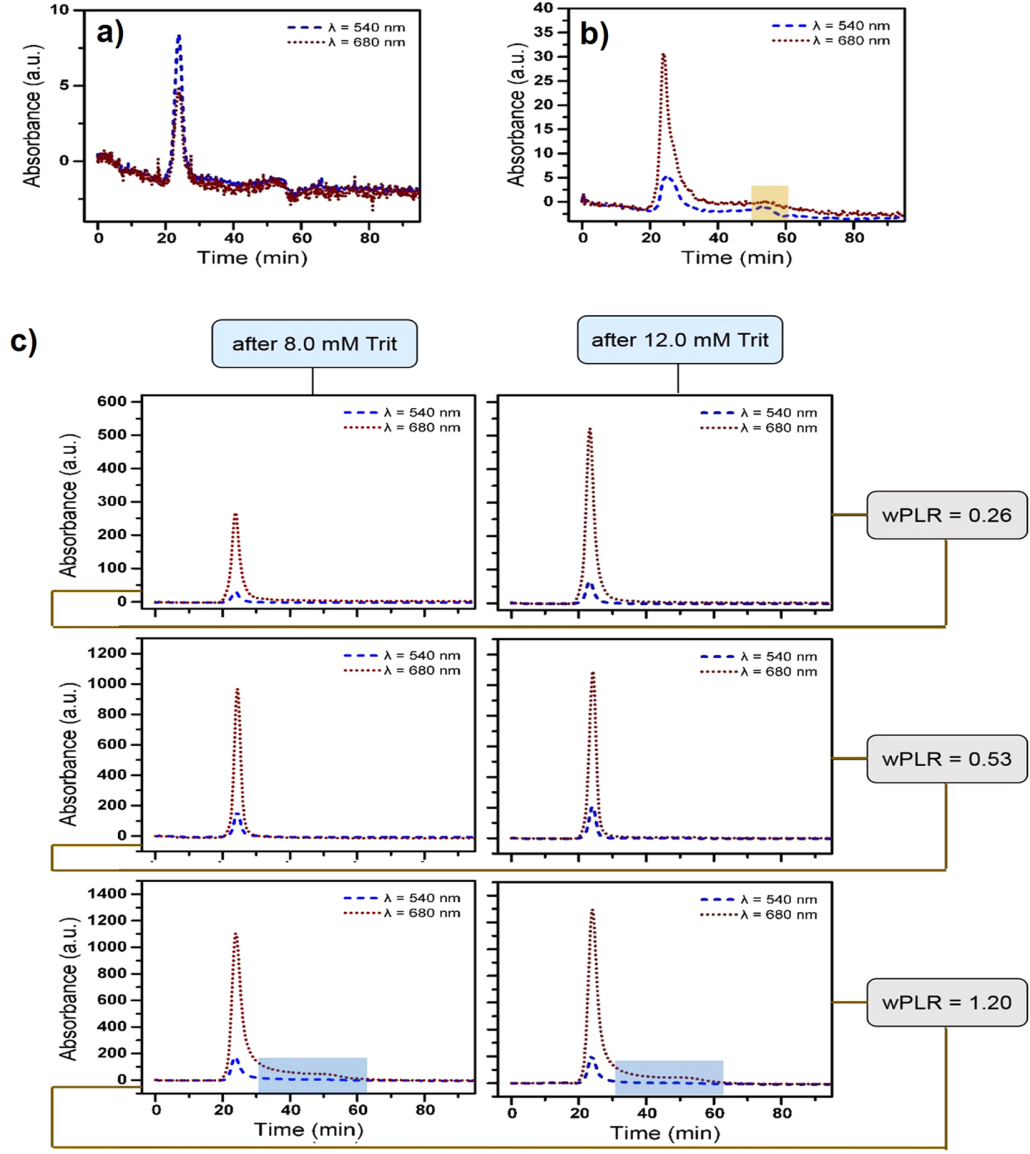
SEC elution profiles of: (**a**) control DPhPG liposomes; (**b**) TX-100 solubilized PSI; and (**c**) PSI-proteoliposomes based on optimal wavelengths of absorbance at 540 (blue dashed lines) and 680 nm (red dot lines) for liposomes and PSI respectively. Shadowed fractions in (**b**) corresponds to detergent micelles. Shadowed fractions in (**c**) for PSI-proteoliposomes at wPLR = 1.2 correspond to individually unassociated PSIs or shredded liposomes.

SEC absorbance profiles of the DPhPG liposomes and TX-100 solubilized PSIs acquired at 540 nm (optimal wavelength for liposome absorption) and 680 nm (optimal wavelength for PSI absorption) provide the controls for the PSI-proteoliposome fractionations and are as shown in Fig. 4a and b respectively. Shadowed fractions in Fig. 4b corresponds to detergent micelles. Comparing the SEC elution profiles of PSI-proteoliposomes with TX-100 solubilized PSIs (680 nm profiles in Fig. 4b) reveals that the PSI-proteoliposomes sample contains no excess detergent micelles due to a lack of an equivalent peak in their chromatogram. Furthermore, SEC elution profiles of PSI-proteoliposomes can be explicitly analyzed from the complete data matrix represented in Fig. 4c for C_Trit_ = 8 and 12 mM (along rows) and wPLR = 0.26, 0.53 and 1.2 (along columns). It is noted that at wPLR = 0.26, choosing a higher detergent concentration within the stage II for protein insertions results in PSI-proteoliposomes with increasing absorbance intensities (~500 a.u.) for 680 nm (Fig. 4c, first row). On the other hand, at wPLR = 0.53, the intensities of 680 nm absorbance almost plateau to a consistent value of ~1000 a.u. for all three protein addition points at C_Trit_ = 8 and 12 mM cases within the stage II of solubilization (Fig. 4c, second row). Finally, at wPLR = 1.2, the absorbance intensities for 680 nm indicate an almost consistent value of ~1200 a.u. for the different C_Trit_ concentrations (Fig. 4c, third row). Here, our hypothesis is that once the first PSI is inserted within the bilayer, it disrupts the membrane integrity which essentially lowers the interfacial free energy for subsequent insertions.

**Figure 5:**
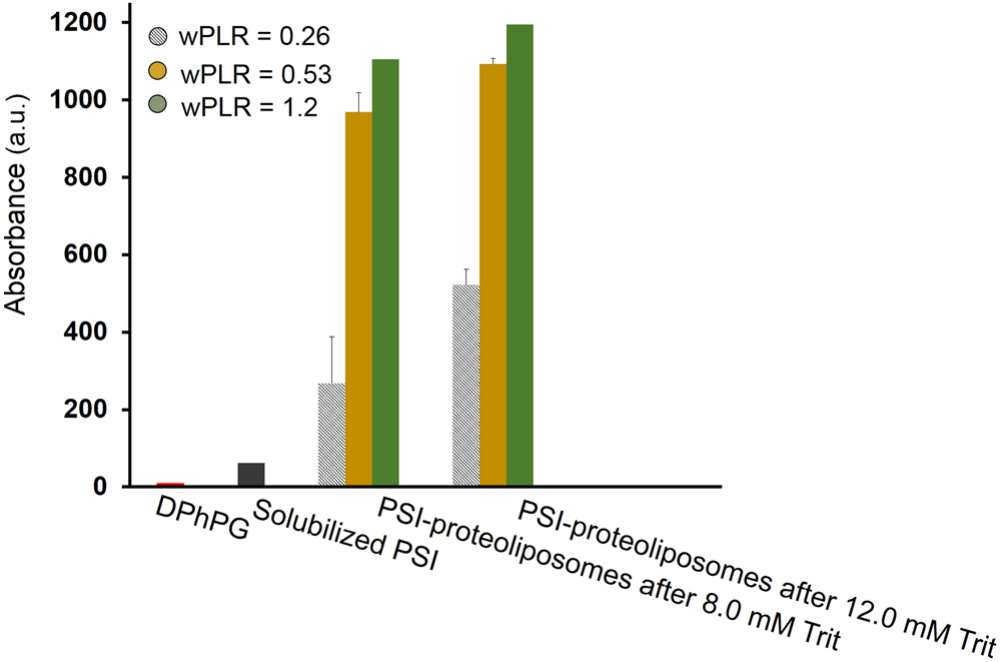
Comparisons of the maximum absorbance intensities at 680 nm as obtained from the SEC elution profiles in Fig. 4.

Preliminary calculations for number of PSI per liposome in the solution results in 1, 2 and 5 PSI(s) per DPhPG liposome for wPLR = 0.26, 0.53 and 1.2 respectively. A careful inspection of these data in regards to the aforementioned SEC absorbance profiles for PSI-proteoliposomes suggest that at wPLR = 0.26, higher detergent concentrations within stage II for protein insertions result in higher number of liposomes carrying one PSI complex within the suspension. However, supplying more PSI proteins to the sample tends to saturate the maximum number of PSIs incorporated within the liposomes. Thus, PSI additions at any point within stage II of solubilization for wPLR = 0.53 and 1.2 results in approximately similar number of liposomes having two or five PSIs respectively. However, the shadowed fractions in the SEC chromatograms of PSI-proteoliposomes at wPLR = 1.2 shows that addition of PSIs at very high concentrations results in PSI-proteoliposomes with higher number of reconstituted PSI as well as individual unassociated PSIs and possibly shredded liposomes present in the sample. Figure 5 shows the relative absorbance intensities at 680 nm for the different PSI-proteoliposomes prepared under different C_Trit_ concentrations and wPLR ratios as derived from the preceding SEC elution steps of purifications. Thus, at wPLR = 1.2, SEC fractions with highest overlapping absorbance intensities at 540 and 680 nm were collected. Afterward, the PSI-proteoliposome formation is characterized mainly by using the intrinsic features of PSI on these fractions.

### Absorption spectroscopy of PSI/DPhPG proteoliposomes

PSI is active over 53% of the solar spectrum with known absorption peaks at around 440 and 680 nm contributed by Chlorophyll *a* (Chl *a*), and shoulders at around 475 and 650 nm originating from Chl *b* in the light-harvesting complexes^60^. Changes in the amplitude and wavelength shifts of absorption peaks of control PSI solution and reconstituted PSI suspension, is a good qualitative indicator of alteration in the microenvironment around the Chl *a* or Chl *b* molecules of PSI. Such qualitative indications of proteoliposome formation has also been demonstrated by earlier studies^61^.

**Figure 6:**
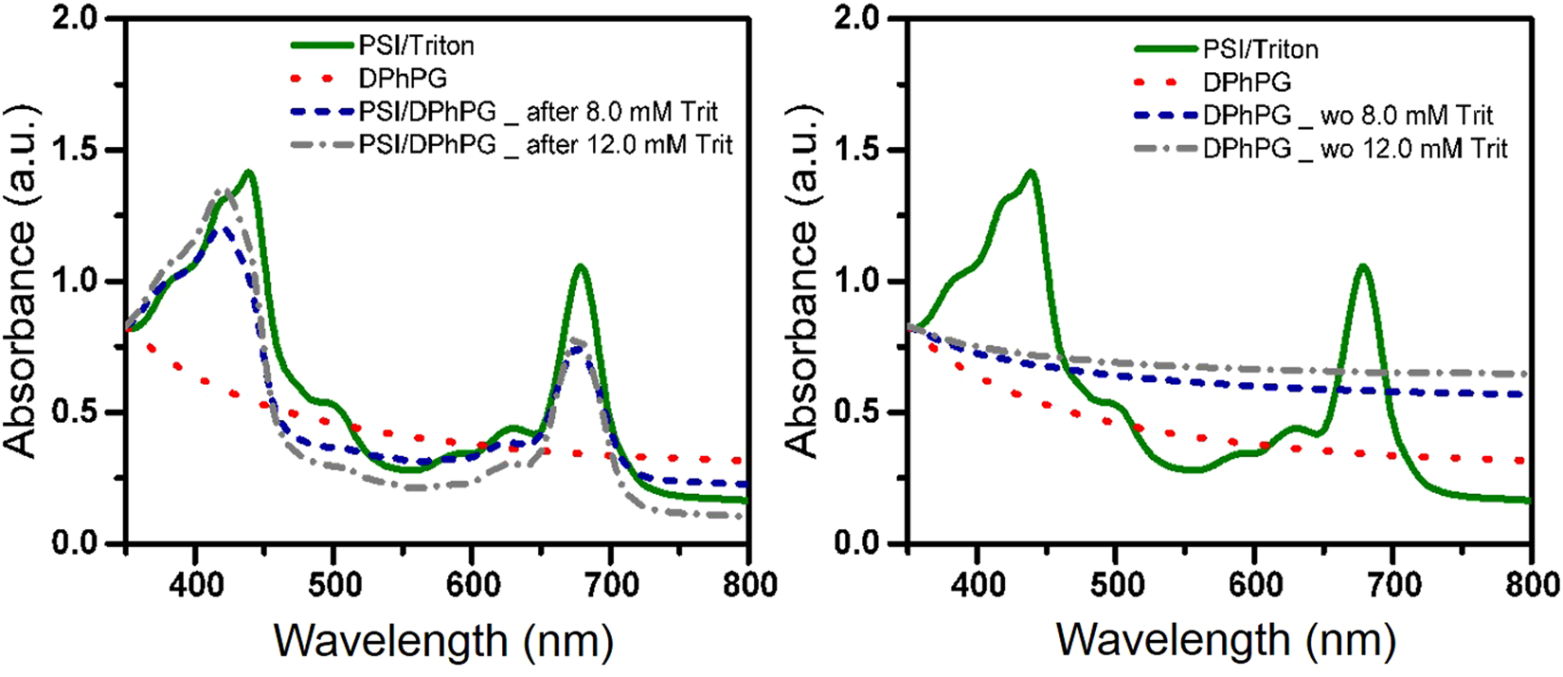
Room temperature absorption spectra of DPhPG liposomes, solubilized PSI and PSI-proteoliposomes with left panel indicating PSI/DPhPG reconstituted at different point of second stage of solubilization and right panel indicating DPhPG liposome suspensions after detergent removal.

The room temperature absorption spectra of TX-100 solubilized PSI solution in Fig. 6 demonstrates the signature peaks of PSI at 440 and 680 nm. The aforementioned peaks are absent in the absorption spectra of control DPhPG suspension. The absorption spectra of PSI-proteoliposome suspension (formed by PSI addition at the aforementioned two points in stage II) shows a significant blue shift in the blue region (peak shift from 440 to 418 nm) and a slight blue shift in the red region (peak shift from 680 to 674 nm). This blue shift is attributed to modification of the microenvironment around Chl *a* molecules within PSI-proteoliposomes (Fig. 6, left panel). Figure 6, right panel shows the room temperature absorption spectra collected for pure DPhPG liposome suspensions devoid of PSI after undergoing identical treatments of detergent removal. The data clearly confirms that the blue shifts are signatures arising from PSI-proteoliposme formations and not an artifact from other microenvironment alterations, such as presence of TX-100 detergent molecules.

### Fluorescence spectroscopy of PSI/DPhPG proteoliposomes

The efficiency of PSI photochemistry is known to benefit from the existence of long-wavelength Chl a molecules (red antennae Chls) that accumulate and stabilize the excitation energy for some period before it is used for the charge separation in P700^62^. The amount of red Chls vary among various species of PSI^58–60^. In particular, cyanobacteria contain relatively high amounts of such red Chls that show absorption at longer wavelengths than the primary Chls of the reaction center itself.

At room temperature, a wide range of the spectra for the absorbed light, including wavelengths longer than 750 nm, offer equally high probability of resulting in a stable P700 photooxidation. On the other side, at low temperatures (1 to 77 K), the red antennae Chls form a deep trap for the excitation energy that promote pathways for a strong increase in the fluorescence yield with red-shifted emission peaks^61–66^. For example, in trimeric PSI-reaction center of *Synechococcus* PCC 6803, the red antennae Chls absorb at ~708 nm and emit at ~721 nm and in the *Spirulina* trimeric PSI-RC, even more red shifted Chls absorb at ~715 nm which give rise to emission bands peaking at ~730 nm at 4 K^67, 68^. In the past, temperature dependence studies for steady-state emission of trimeric PSI of *Synechococcus elongatus* and quantum yield of P700 oxidation have showed that upon cooling from room temperature to ultra-low temperature (5 K), the emission peak wavelength shifts from 721 nm to 732 nm^63^. This study suggests that at 5 K almost 50% of the nonselective excitation energy becomes irreversibly trapped with the red antennae Chls, and hence, a considerable part of the excitation energy does not result in P700 photo-oxidation.

Moreover, detailed analysis of fluorescence emission peaks can provide complementary qualitative methods for the indirect evaluation of successful proteoliposome formations^63^. To this end, it has been frequently suggested by earlier studies that the appearance of an emission peak at 680 nm can be indicative of uncoupled Chls^61, 64, 65^ which is typically considered as a marker for proteoliposome formations.

**Figure 7:**
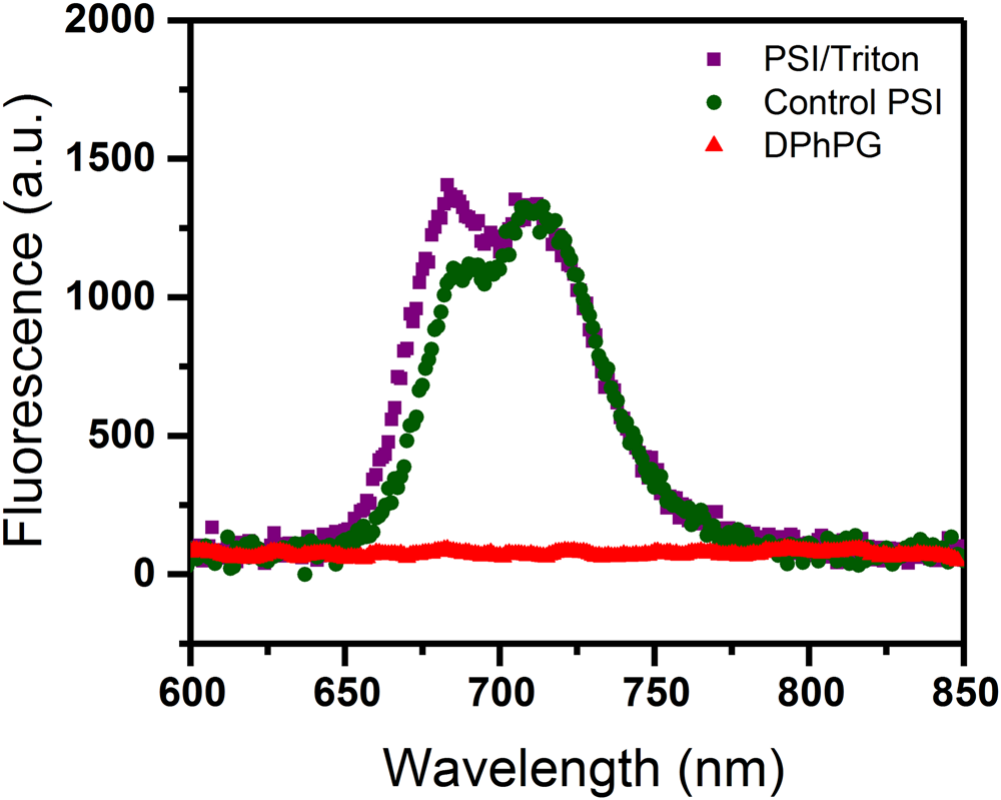
Room temperature fluorescence emission spectra of DPhPG liposomes, TX-100 solubilized PSI, and control PSI suspensions.

**Figure 8:**
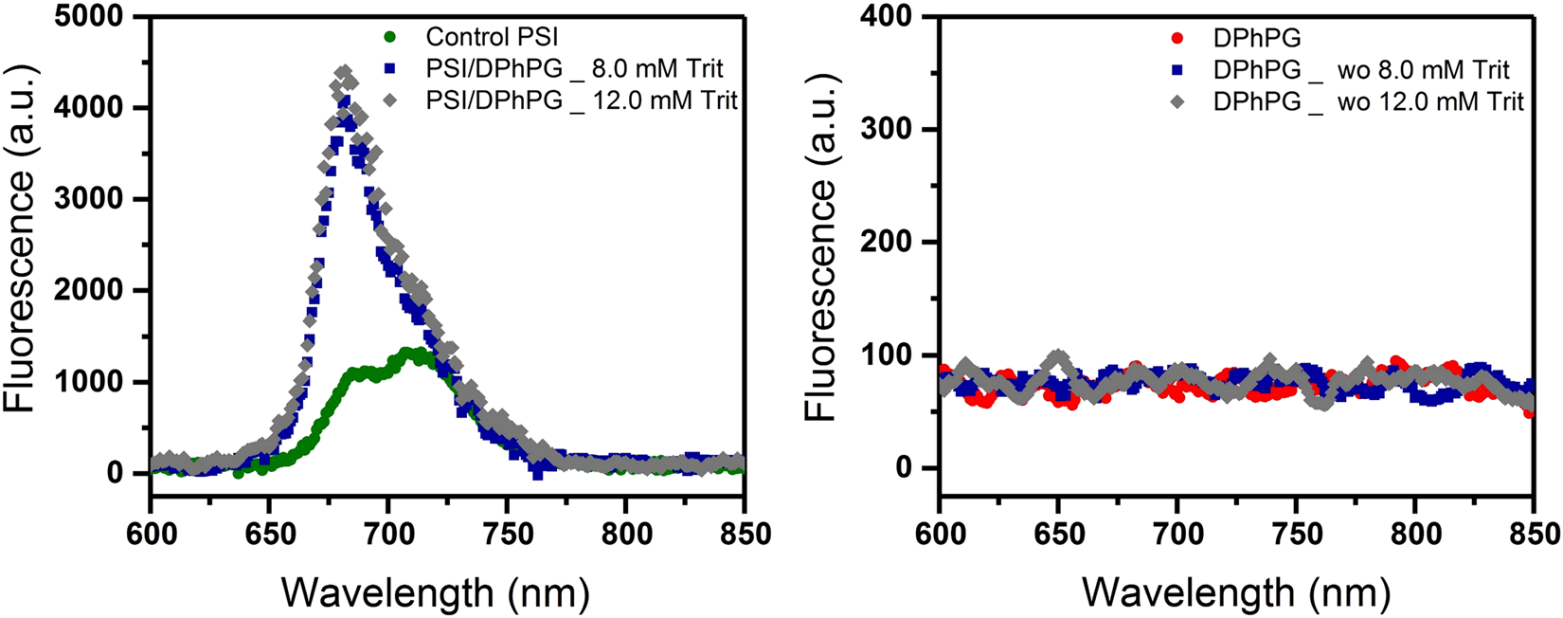
Room temperature fluorescence emission spectra of: (left panel) control PSI and PSI-proteoliposomes reconstituted at different point of second stage of solubilization; (right panel) control DPhPG liposomes and DPhPG liposomes suspension after detergent removal.

In the present study, fluorescence emission spectra of control PSI (without detergent), PSI/detergent solutions as well as pure DPhPG suspensions upon excitation at 440 nm in room temperature conditions are shown in Fig. 7. Fluorescence emission spectra of PSI solution in the absence of detergents indicate two emission bands at around 690 nm and 712 nm (1100 and 1300 fluorescence counts, respectively). Fluorescence emission spectra of PSI solution, in the presence of detergents, indicate a similar emission band at 712 nm but a blue-shifted and enhanced emission band at ~685 nm (both ~1300 fluorescence counts). As expected, the emission band at 690 nm is not observed in the case of the pure liposome suspensions. After PSI incorporation, the proteoliposomes were further characterized by fluorescence emission spectroscopy at room temperature as shown in Fig. 8. Figure 8 (left panel) shows florescent emissions from proteoliposome suspensions after PSI reconstitution into detergent-destabilized DPhPG liposomes. Both the emission spectra for PSI-proteoliposomes formed from protein insertions at the intermediate (C_Trit_ ~ 8 mM) and final (C_Trit_ ~ 12 mM) states in stage II of solubilization indicate ~4 fold enhancements in their fluorescence intensities at the single dominant peak of 680 nm as compared to the control PSI case (see Fig. 8). Such enhanced fluorescence intensity at 680 nm observed for the TX-100 mediated proteoliposome suspension further confirms the successful proteoliposome formation. Furthermore, the lack of a 680 nm peak in the fluorescence emission spectra for DPhPG liposome suspensions after undergoing identical treatments for detergent removal (Fig. 8, right panel) further corroborates our hypothesis that observed Chl uncoupling is due to the proteoliposome formations. It needs to be pointed out here that the red shifted emission peaks at ~712 nm were not observed in the case of proteoliposome suspensions, which clearly indicates a higher amount of excitation energy being funneled towards P700 photooxidation instead of being lost as fluorescence emissions in the case of PSI devoid of membrane confinements. Thus, the aforementioned observations with PSI confinements in synthetic lipid scaffolds provide evidences that membrane confinements, beyond acting as the protein structural scaffolding, play a functional role in tuning the photoresponse in PSI-proteoliposome systems.

### Atomic force microscopy of PSI/DPhPG proteoliposomes

**Figure 9:**
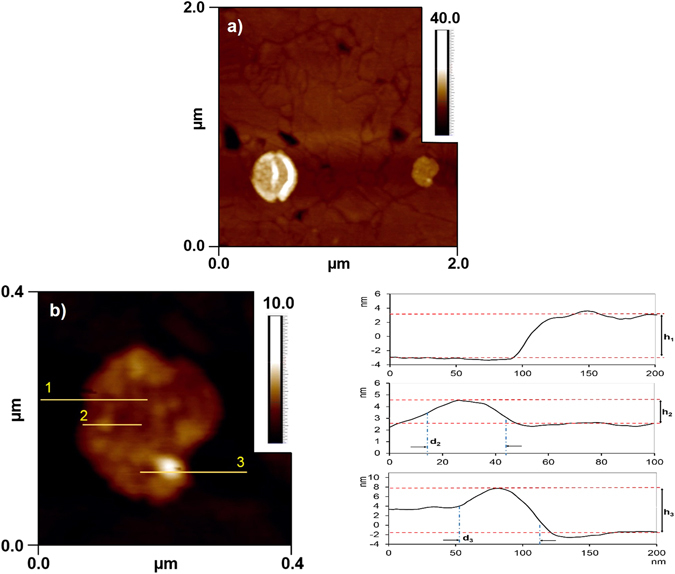
AFM images of the PSI-proteoliposomes supported on gold surfaces. (**a**) Bare gold substrate and PSI-proteoliposomes; (**b**) zoomed in images of PSI-proteoliposomes and representative cross-sectional surface profiles (1) for lipid bilayer assembled on gold surface (h_1_ ~ 5 nm); (2) for membrane-bound PSIs (h_2_ ~ 2 nm, d_2_ ~ 30 nm) and (3) for membrane-adsorbed PSI (h_3_ = 8.5 nm, d_3_ = 50 nm) are shown for the respective AFM image.

A droplet of suspension after SEC column was drop cast on the gold wafers as mentioned in the methods section. AFM topographical characterization of this sample further confirms the successful PSI-proteoliposome formation. For ease of visualization, highly dilute samples from the SEC elution profiles were drop cast on to the substrates to create sparsely located supported collapsed bilayers. The AFM image of suspension treated gold substrate depicted in Fig. 9a shows two immobilized liposomes on the Au surface and barely any individual PSI. Zoomed-in AFM images in Fig. 9b clearly show serval membrane-bound PSIs as well as membrane-adsorbed PSIs as also indicated from the SEC elution profiles for the case studies with wPLR = 1.2 in Fig. 4c.

Detailed cross-sectional surface profiles from the aforementioned AFM image of supported liposomes have been reported in Fig. 9b, right panel. The cross-sectional profiles indicated an average bilayer height of h_1_ = 5 nm (shown with arrows marked on Fig. 9b) as expected for the lipid bilayer system. Furthermore, surface profiles of the immobilized liposome show an average height of h_2_ = 2 nm as well as average dimeter of d_2_ = 30 nm, which represent membrane-bound PSI and an average height of h_3_ = 8.5 nm as well as average dimeter of d_2_ = 50 nm, which represent membrane-adsorbed PSI (also indicated in Figure S1, Supplementary Material).

## Discussion

Considering the large negatively charged PG content in thylakoid membrane of PSI, we aim to introduce a choice of PG lipid for the study of reconstituted PSI proteins and subsequently systematically incorporate PSI complexes into synthetic membrane-bound structures that mimic the natural thylakoid membrane housing of PSI. Specifically, the current study presents the surfactant-induced membrane solubilization of a negatively charged phospholipid DPhPG by TX-100 commonly used for protein solubilization. Our results indicate that DPhPG liposomes maintain their vesicular structures intact at the initial stages of detergent addition. Furthermore, when solubilized with TX-100, the vesicles undergo the typical three-stage transition to the final micellar stages via mixed vesicles-micelles state. Based on the phase diagram obtained from the solubilization analysis, ideal concentration ratios for successful protein insertion were chosen and PSI complexes were incorporated into detergent-destabilized preformed-liposomes at various points of stage II of solubilization, using polystyrene beads to adsorb the detergent. Successful PSI-proteoliposome formation was analyzed via absorption and fluorescence spectroscopy measurements and atomic force microscopy.

A clear signature for successful PSI-proteoliposome formation is observed when a significant blue shift in the blue region (peak shift from 440 to 418 nm) and a slight blue shift in the red region of absorbance spectra and enhanced fluorescence intensity at 680 nm is observed for the TX-100 mediated proteoliposome suspension. AFM topographical characterization of supported liposomes revealed serval membrane-bound PSIs as well as membrane-adsorbed PSIs, further stablishing the successful PSI-proteoliposome formation. Such observations throw light on the alterations in chromophore-chromophore interactions and Chl *a* photoexcitation (as indicated by the enhanced 680 nm fluorescence emissions) owing to microenvironment changes arising from PSI confinement within fabricated proteoliposome structures. In turn, the current study paves the path for optimal tuning of photochemical responses of PSI complexes through rationally designed synthetic bilayer membrane confinements that mimic the natural thylakoid membrane housing of PSI.

## Experimental Section

### Materials

Dibasic (Na_2_HPO_4_) and monobasic (NaH_2_PO_4_) sodium phosphate with > 99% assay were purchased from Fisher Scientific, were used to prepare the aqueous buffer solutions of 200 mM Na-Phosphate with pH = 7.0. All aqueous buffer solutions of 200 mM Na-Phosphate were prepared in ultrapure de-ionized (D.I.) water with a resistivity of 18.2 MΩ cm at 25 °C (Millipore, Billerica, MA). Triton X-100 (10% w/v aqueous solution) was obtained from Anatrace. DPhPG (1,2-diphytanoyl-*sn*-glycero-3-phospho-(1′-*rac*-glycerol)) was purchased as lyophilized powders from Avanti Polar Lipids, Inc. Polycarbonate filters were also purchased from Avanti Polar Lipids, Inc. Lacey carbon-coated (200 mesh) copper grids were purchased from SPI supplies, USA.

## Methods

### Growth of *T*. *elongatus* and preparation of Photosystem I

The thermophilic cyanobacterium *T*. *elongatus* BP-1 was grown in 2 L airlift fermenters (Bethesda Research Labs, Bethesda MD) in NTA media. The details for the extraction and purification of the trimeric PSI complex from the grown *T*. *elongatus* cells are provided elsewhere.[21] Based on spectrophotometer measured chlorophyll concentrations, the concentration of the extracted PSI trimers was estimated to be around 1.0 × 10^−6^ mol L^−1^. PSI trimers were stored in aliquots of 1.5 mL at −80 °C for future use.

### Liposome preparation

4 mg ml^−1^ lipid suspensions were prepared in 200 mM Na-Phosphate (pH = 7.0) buffer, followed by 3–4 freeze–thaw cycles to form multilamellar liposomes. These suspensions were then extruded through 100 nm pore sized filter using a mini-extruder (Avanti Polar Lipids) to form unilamellar vesicles at room temperature. The large unilamellar vesicle size of ~100 nm was confirmed from dynamic light scattering measurements. Further details regarding the lipid vesicle preparations can be found in previous literatures^69^.

### Titrations of liposomes with detergent

Liposomes suspensions of 4 mg ml^−1^ were titrated by stepwise addition of several aliquots of 10% wt vol^−1^ of TX-100 to the liposome suspension. Detailed technical specifications regarding the physical properties of TX-100 are provided in Table S1 of Supplementary Material^50, 70, 71^. The effect of detergent on the liposomes was monitored using isothermal titration calorimetry.

### Isothermal titration calorimetry (ITC)

A VP-ITC instrument produced by MicroCal Inc. (Northampton, MA) was used. The cell (volume 1.4 ml) was filled with 1 mM or 4 mM DPhPG vesicle suspension. The injection syringe was filled with 300 *μ*l of 77 mM or 154 mM detergent solution, and a series of different volumes of injections were made at 30 min intervals. During each injection, surfactant was incorporated into the lipid membranes, leading to a characteristic heat signal. Integration of the signal yielded the heat change for each injection. The detailed ITC procedure of TX-100 mediated solubilization of DPhPG is provided in Supplementary Material.

### Dynamic light scattering (DLS)

To analyze the liposome size alterations induced by the interaction with the detergent molecules, dynamic light scattering measurements (DLS) were carried out using a 632.8 nm-wavelength Zetasizer (Malvern Instruments). All DLS data were collected using a 178° backward scattering and averaged over four experimental runs each of which were summed up over 12 time correlograms fitted by the Zetasizer software. Due to the presence of bimodal or multimodal size distributions in some phase stages of the solubilization process, the DLS data are represented by more than one curve. All reported sizes are in terms of equivalent spherical hydrodynamic radius as estimated from Stokes–Einstein relation, and all DLS analyses are represented in terms of number distribution. The data analysis was carried out using the effective thermo-physical properties of 200 mM Na-Phosphate aqueous buffer solutions with pH = 7.0.

### Cryo-transmission electron microscopy (Cryo-TEM)

Cryo-TEM allows direct investigation of samples in their vitrified state at low temperature. The sample preparation for cryo-TEM technique was carried out on 200-mesh carbon-coated holey grids. Before the sample application, a glow discharge was performed in order to hydrophilize the grid for optimal spreading of the aqueous sample^72^. The suspension was then drop cast onto TEM grids following which the excess sample was absorbed using a filter paper, leaving a thin film of sample in the holes of the grid. Then, the grid was mounted in a Gatan cryo-plunge 3 device and immediately frozen by plunging it in liquid ethane cooled by liquid nitrogen. After vitrification, the frozen-hydrated specimen was inserted into the Gatan cryo-transfer system and transferred into the TEM system. The imaging was carried out by a Zeiss Libra 200 MC TEM equipped with a model Gatan 915 cryo-specimen holder, at an acceleration voltage of 200 kV, and a temperature of about −170 °C under strict low dose conditions (<15 e Å^−2^). Images were recorded with a Gatan UltraScan 1000XP. Quantitative analyses on more than *n* >30 particles were used to measure the particle size at each stage of solubilization.

### Reconstitution of PSI

Based on ITC measurements and phase diagram appropriate amounts of TX-100 were calculated to solubilize the DPhPG within stage II of solubilization. Subsequently, detergent-mediated protein reconstitution was performed by addition of TX-100 to preformed liposomes (4 mg mL^−1^). The solution was equilibrated for 60 min, mixed with the solubilized protein in 200 mM Na-Phosphate buffer (pH 7.0), and afterward, the mixture was incubated for 30 min at room temperature under gentle agitation. The final protein-to-lipid weight ratios used for all our studies here (marked as wPLR in all our discussions) were 0.26, 0.53 and 1.2. For PSI reconstitution, TX-100 was removed by a slow removal procedure in two successive steps, namely, additions of 15 mg Bio-Beads per milligram of TX-100 for 1 h at room temperature and 12 h at 4 °C, followed by an addition of 15 mg Bio-Beads per milligram of TX-100 at room temperature to ensure full detergent removal.

### Size-exclusion chromatography (SEC)

SEC separations were carried out using a Sephacryl S-400 column attached to an AKTA purifier (GE). A total of 200 μl of sample was loaded at a flow rate of 0.5 ml min^−1^. The sample elution was monitored by optimal wavelength for liposome and PSI absorbance at 540 and 680 nm, thereby enabling the detection of both PSI and lipids.

### Absorption spectroscopy measurements

Absorption spectra were recorded at room temperature with a hybrid multi-mode microplate reader (Make: Biotek; Model: Synergy H1). The incubation media contained 200 mM Na-Phosphate at pH 7.0. Absorption spectra for PSI and proteoliposome suspensions were specifically monitored at ~440 and 680 nm (details discussed in results section).

### Fluorescence measurements

Room temperature fluorescence spectra of the samples were obtained using a multi-mode microplate reader (Make: PerkinElmer; Model: EnSpire) with 440 nm as the excitation wavelength. The fluorescence emission spectra were measured between 600 and 850 nm.

### Atomic force microscopy (AFM)

AFM topographical characterization for PSI-proteoliposome Au substrates was carried out on commercial gold coated silicon wafers, Au thickness ~100 nm (purchased from Platypus Technologies). Gold wafers were cleaned by immersion in isopropanol (99.99% v/v) and de-ionized water for 10 min, and drying in an N_2_ stream. Surface immobilization of PSI-proteoliposomes was carried out by drop casting a few microliters of PSI-proteoliposome suspension after size exclusion chromatography on the Au wafers. The monolayer-covered gold wafer was allowed to dry for 1 h at 158 F and 1 h at room temperature and then rinsed in de-ionized water and dried in an N_2_ stream. Surface topography images were collected on an AFM instrument (NT-MDT) in the tapping mode using a silicon cantilever compatible with softer biological materials (NT-MDT; Model: NSG03). The tip had a force constant of 0.35–6.1 N m^−1^ along with a resonant frequency of 47–150 kHz.

## Electronic supplementary material

Supplementary Material
