# Supplementary material for: Tuning the photoexcitation response of cyanobacterial Photosystem I via reconstitution into Proteoliposomes
Source: Sci Rep. 2017 May 30;7:2492. doi: 10.1038/s41598-017-02746-5 (PMC5449388; doi:10.1038/s41598-017-02746-5)
Supplement: Supplementary file 1 — Supplementary Material [file 41598_2017_2746_MOESM1_ESM.pdf]

Tuning the photoexcitation response of cyanobacterial Photosystem I via reconstitution into Proteoliposomes

Hanieh Niroomand,[†,#], Dibyendu Mukherjee,[*,†,#,‡], and Bamin Khomami[*,†,#,‡]

[†]Sustainable Energy Education and Research Center (SEERC); [#]Department of Chemical and Biomolecular Engineering; [‡]Department of Mechanical, Aerospace and Biomedical Engineering, University of Tennessee, Knoxville

Corresponding Author:

Bamin Khomami; Phone: (865) 974-2421; Fax: (865) 974-7076; E-mail: bkhomami@utk.edu

Dibyendu Mukherjee; Phone: (865) 974-5309; Fax: (865) 974-5274; E-mail: dmukherj@utk.edu

Supplementary Note.

Monitoring TX100-mediated solubilization of DPhPG with isothermal titration calorimetry (ITC).

TX100-mediated solubilization of DPhPG was monitored by following the Nature protocol pioneered by Heerklotz et al.[1] The detailed procedure is as follow:

For the first solubilization experiment, the 1 mM lipid suspension and the 77 mM detergent solution were brought to a temperature slightly below the desired experimental temperature (23–24 °C) and degassed by stirring the open vials under reduced pressure for ~10 min. The calorimeter cell was filled with the lipid suspension and the syringe with the detergent solution. The net volumes are ~1.4 ml for the cell and ~300 µl for the syringe. In the driver software of the calorimeter, the experimental parameters such as temperature, concentrations, stirring speed, reference power, feedback mode, number of injections, injection volumes, injection speeds, spacings, etc were specified. 35 injections were performed with spacing of 30-40 minutes. A sequence 5 × 2.0, 1.5, 1.5, 2.0, 2.0, 3.0, 3.0, 4.0, 8.0, 5.0, 6.0, 7.0, 7.0, 5.6, 8.3, 8.9, 9.7, 10.5,11.3, 12.3, 13.3, 14.3, 14.3, 15.0, 15.0, 15.0, 16.5, 20.0, 20.0, 10.0 and 20.0 µl volumes in the course of the titration was used.

For the second solubilization experiment, the 4 mM lipid suspension and the 154 mM detergent solution were brought to a temperature slightly below the desired experimental temperature (23–24 °C) and degassed by stirring the open vials under reduced pressure for ~10 min. The calorimeter cell was filled with the lipid suspension and the syringe with the detergent solution. The net volumes are ~1.4 ml for the cell and ~300 µl for the syringe. 41 injections were performed with spacing of 30-40 minutes. A sequence 2.0, 4 × 3.0, 4.0, 2.0, 3.0, 3.0, 2.0, 4 × 4.0, 2.0, 3.0, 4.0, 3.0, 4.0, 3 × 3.0, 7.0, 4.0, 5.0, 5.0, 6.0, 4.0, 6.0, 4.0, 7.0, 6.0, 5.0, 7.0, 13.0, 10.0, 20.0, 22.0, 25.0, 25.0, 30.0 µl volumes in the course of the titration was used.

Supplementary Note.

Phase diagram explanation.

The phase diagram is established based on the two series of solubilization experiments starting at different lipid concentrations (1 mg ml^{-1} and 4 mg ml^{-1}).

As the phase boundaries obey, in the ideal case, linear fits of the two boundaries based on the equations

$$C_D^{SAT}(C_L) = R_e^{SAT} C_L + C_D^{aq,SAT} = R_e^{SAT} (C_L + \frac{1}{KR}) \quad (1)$$

and

$$C_D^{SOL}(C_L) = R_e^{SOL} C_L + C_D^{aq,SOL} = R_e^{SOL} (C_L + \frac{CMC}{1+ReSOL}) \quad (2)$$

would provide the maximal detergent-to-lipid mole ration in membranes (onset of solubilization), R_e^{SAT}, the minimal detergent-to-lipid mole ratio in micelles (completion of solubilization), R_e^{SOL} and the free detergent concentration on solubilization.

Intercept of the linear fits of the two boundaries give

$C_D^{SAT}(C_L) = 0.67\ C_L + 1.05$

and

$C_D^{SOL}(C_L) = 3.17\ C_L + 1.05$

where $C_D^{aq,SAT} = C_D^{aq,SOL} = 1.05$ mM, the CMC of the pure TX-100 is 1.38 mM and the membrane–water partition coefficient (K_R) is 0.64 mM^{-1}.

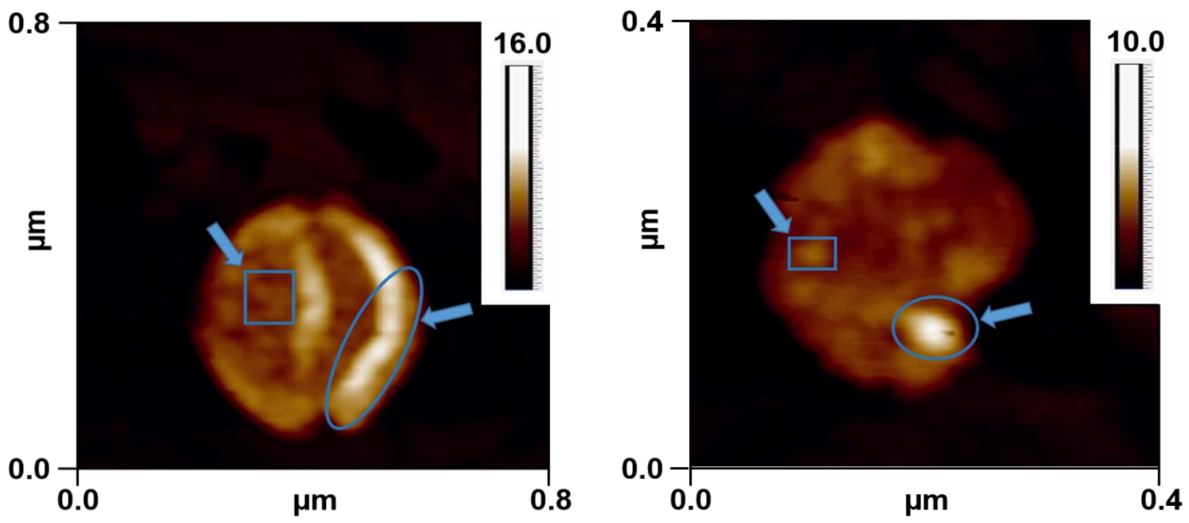

Figure S1. Zoomed-in AFM images of PSI-proteoliposomes on gold surface. Membrane-bound PSIs and membrane-adsorbed PSIs are shown.

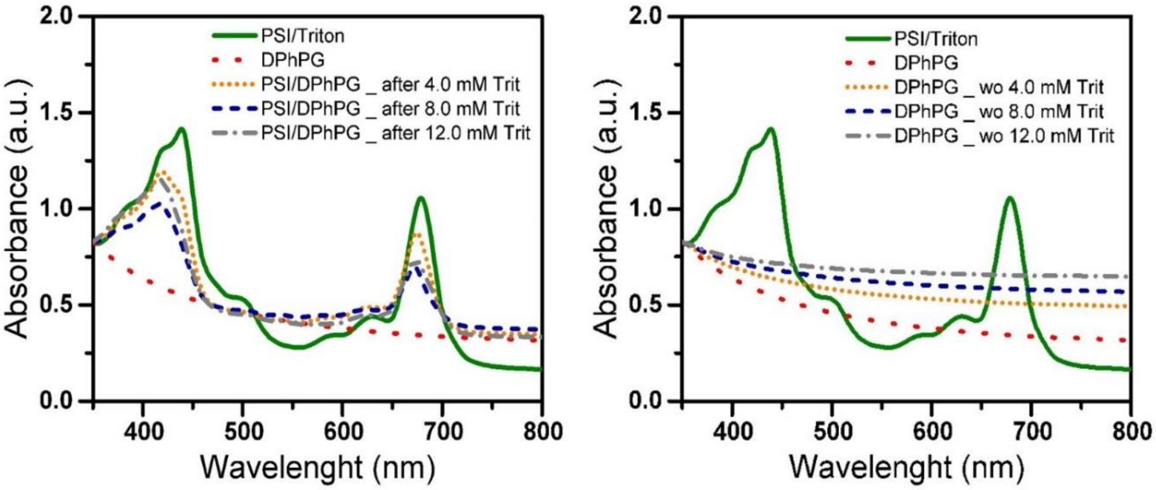

Figure S2. Room temperature absorption spectra of DPhPG liposomes, solubilized PSI and PSI-proteoliposomes of: (left panel) PSI-proteoliposomes; (right panel) DPhPG liposomes suspension after detergent removal.

Table S1. Physical properties of the detergents, TX-100

	MW	Aggregation Number	Micellar Weight (KD)	CMC (mM)
Triton X-100	625	73-140	46-90	0.21

[1] H. Heerklotz, A.D. Tsamaloukas, S. Keller, Monitoring detergent-mediated solubilization and reconstitution of lipid membranes by isothermal titration calorimetry, Nat. Protocols, 4 (2009) 686-697.
